# The Theory of Localist Representation and of a Purely Abstract Cognitive System: The Evidence from Cortical Columns, Category Cells, and Multisensory Neurons

**DOI:** 10.3389/fpsyg.2017.00186

**Published:** 2017-02-16

**Authors:** Asim Roy

**Affiliations:** Department of Information Systems, Arizona State University, TempeAZ, USA

**Keywords:** localist representation, distributed representation, amodal representation, abstract cognitive system, theory of the brain, cortical columns, category cells, multisensory neurons

## Abstract

The debate about representation in the brain and the nature of the cognitive system has been going on for decades now. This paper examines the neurophysiological evidence, primarily from single cell recordings, to get a better perspective on both the issues. After an initial review of some basic concepts, the paper reviews the data from single cell recordings – in cortical columns and of category-selective and multisensory neurons. In neuroscience, columns in the neocortex (cortical columns) are understood to be a basic functional/computational unit. The paper reviews the fundamental discoveries about the columnar organization and finds that it reveals a massively parallel search mechanism. This columnar organization could be the most extensive neurophysiological evidence for the widespread use of localist representation in the brain. The paper also reviews studies of category-selective cells. The evidence for category-selective cells reveals that localist representation is also used to encode complex abstract concepts at the highest levels of processing in the brain. A third major issue is the nature of the cognitive system in the brain and whether there is a form that is purely abstract and encoded by single cells. To provide evidence for a single-cell based purely abstract cognitive system, the paper reviews some of the findings related to multisensory cells. It appears that there is widespread usage of multisensory cells in the brain in the same areas where sensory processing takes place. Plus there is evidence for abstract modality invariant cells at higher levels of cortical processing. Overall, that reveals the existence of a purely abstract cognitive system in the brain. The paper also argues that since there is no evidence for dense distributed representation and since sparse representation is actually used to encode memories, there is actually no evidence for distributed representation in the brain. Overall, it appears that, at an abstract level, the brain is a massively parallel, distributed computing system that is symbolic. The paper also explains how grounded cognition and other theories of the brain are fully compatible with localist representation and a purely abstract cognitive system.

## Introduction

We have argued for decades about how features of the outside world (both abstract and concrete) are encoded and represented in the brain (Newell and Simon, 1976; Newell, 1980; Smith, 1982; Hinton et al., 1986; Earle, 1987; Smolensky, 1987, 1988; Fodor and Pylyshyn, 1988; Rumelhart and Todd, 1993). In the 70s and 80s, however, when the various theories were proposed and most of the fundamental arguments took place, study of the biological brain was still in its infancy. We, therefore, didn’t have much neuroscience data to properly evaluate the competing theories. Thus, the arguments were mainly theoretical. Fortunately, that situation has changed in recent years with a significant amount of research in neurophysiology. We are, therefore, in a better position now to evaluate the competing theories based on real data about the brain.

Freeman and Skarda (1990) have argued that the brain does not need to encode or represent features of the outside world in any explicit way. Representation, however, is a useful abstraction for computer and cognitive sciences and for many other fields and neurophysiology continues to search for correlations between neural activity and features of the external world (Logothetis et al., 1995; Chao and Martin, 2000; Pouget et al., 2000; Freedman et al., 2001; Wang et al., 2004; Quiroga et al., 2005; Samejima et al., 2005; Averbeck et al., 2006; Martin, 2007; Patterson et al., 2007; Kriegeskorte et al., 2008). In fact, the two Nobel prizes in physiology for ground-breaking discoveries about the brain have been about encoding and representation: (1) Hubel and Wiesel’s discovery of a variety of fundamental visual processing cells in the primary visual cortex, such as line, edge, color and motion detector cells (Hubel and Wiesel, 1959, 1962, 1968, 1977), and (2) the discovery of place cells by O’keefe and grid cells by Mosers (O’Keefe and Dostrovsky, 1971; O’keefe and Nadel, 1978; Moser et al., 2008). Thus, in this paper, I focus primarily on the two main competing theories of representation – localist vs. distributed.

The cortical column – a cluster of neurons that have similar response properties and which are located physically together in a columnar form across layers of the cortex – is now widely accepted in neuroscience as the fundamental processing unit of the neocortex (Mountcastle, 1997; Horton and Adams, 2005; DeFelipe, 2012). There are some very interesting findings from studies of the cortical columns and it makes sense to understand the nature and operation of cortical columns from a representational and computational point of view. So that is a major focus of this paper.

Encoding of complex abstract concepts is the second major focus of this paper. Distributed representation theorists have always questioned whether the brain is capable of abstracting complex concepts and encoding them in single cells (neurons) or in a collection of cells dedicated to that concept. There was an article in *MedicalExpress* (Zyga, 2012) on localist representation following the publication of Roy (2012). That article includes an extensive critique of localist representation theory by James McClelland. I quote here a few of his responses regarding encoding of complex concepts:

(1) “*what basis do I have for thinking that the representation I have for any concept – even a very familiar one – as associated with a single neuron, or even a set of neuronsdedicated only to that concept*?”
(2) “*A further problem arises when we note that I may have useful knowledge of many different instances of every concept I know – for example, the particular type of chicken I purchased yesterday evening at the supermarket, and the particular type of avocados I found to put in my salad. Each of these is a class of objects, a class for which we may need a representation if we were to encounter a member of the class again. Is each such class represented by a localist representationin the brain*?”

As one can sense from these arguments, the nature and means of encoding of complex abstract concepts is a major issue in cognitive science. A particular type of complex abstract concept is the concept of a category. There are several neurophysiological studies on category representation in the brain and they provide some new insights on the nature of encoding of abstract concepts. I review some of those studies that show that single cells can indeed encode abstract category concepts.

I also address the issue of modality-invariant (or amodal) representation, which is also a form of abstraction, and provide evidence for the extensive use of an amodal cognitive system in the brain where such abstractions are encoded by single cells. Finding these different kinds of abstractions in the brain (from categorization to modality-invariance) resolves a long standing dispute within cognitive science – between grounded cognition, which is modality-based, and the traditional cognitive system defined on the basis of abstractions (Borghi and Pecher, 2011). Given the evidence for grounded cognition (Barsalou, 2008) and the various forms of abstractions encoded by single cells, it is fair to claim that both a purely abstract form of cognition and modality-dependent cognition co-exist in the brain providing different kinds of information and each is supported by localist representation.

Finally, I address the issue of distributed representation or population coding (Panzeri et al., 2015) and its conflict with the evidence for localist representation. I essentially argue that there is no evidence for distributed representation because there is no evidence for dense distributed coding. And dense distributed coding is the essential characteristic of distributed representation as claimed by some of the original proponents (McClelland et al., 1995).

The paper has the following structure. In Section “Localist vs. Distributed Representation,” I provide the standard definitions for localist and distributed representations and explain the difference between distributed processing and distributed representation. In Section “Columnar Organization in the Neocortex,” I explore the neuroscience of columnar organization in the neocortex and what it implies for representational theories. In Section “Category Cells,” I review neurophysiological studies that relate to encoding of category concepts in the brain. Section “Multisensory Integration in the Brain” has the evidence for multi-sensory integration and modality-invariant single cells in the brain. In Section “The Existence of a Single Cell-Based Purely Abstract and Layered Cognitive System and Ties to Grounded Cognition,” I argue that there’s plenty of evidence for a purely abstract, single-cell based cognitive system in the brain. In addition, I argue that a sensory-based (grounded) non-abstract and a purely abstract cognitive system co-exist and support each other to provide cognition in its various forms. In Section “On the “Meaning and Interpretation” of Single Neuron Response,” I explain what “meaning and interpretation” implies for a single cell response. Section “Localist Representation and Symbols” explains why localist neurons are symbols in a computational and cognitive sense. Section “No Evidence for Distributed Representation” argues that there is no neurophysiological evidence for distributed representation because distributed representation is about dense representation. Section “Conclusion” has the conclusions.

## Localist vs. Distributed Representation

### Definitions and What They Mean

Distributed representation is generally defined to have the following properties (Hinton et al., 1986; Plate, 2002):

• A concept is represented by a pattern of activity over a collection of neurons (i.e., more than one neuron is required to represent a concept).
• Each neuron participates in the representation of more than one concept.

By contrast, in localist representation, a single neuron represents a single concept on a stand-alone basis. But that doesn’t preclude a collection of neurons representing a single concept. The critical distinction between localist units and distributed ones is that localist units have “meaning and interpretation” whereas the distributed ones don’t. Many authors have pointed out this distinction.

• Elman (1995, p. 210): “*These representations are distributed, which typically has the consequence that interpretable information cannot be obtained by examining activity of single hidden units*.”
• Thorpe (1995, p. 550): “*With a local representation, activity in individual units can be interpreted directly… with distributed coding individual units cannot be interpreted without knowing the state of other units in the network*.”
• Plate (2002):“*Another equivalent property is that in a distributed representation one cannot interpret the meaningof activity on a single neuron in isolation: the meaning ofactivity on any particular neuron is dependent on the activityin other neurons (Thorpe, 1995).*”

Thus, the fundamental difference between localist and distributed representation is only in the interpretation and meaning of the units, nothing else. Therefore, any and all kinds of models can be built with either type of representation; there are no limitations as explained by Roy (2012).

Reviewing single cell studies, Roy (2012) found evidence that single cell activations can have “meaning and interpretation,” starting from the lowest levels of processing such as the retina. Thus, localist representation is definitely used in the brain. Roy (2013) found that multimodal invariant cells exist in the brain that can easily identify objects and concepts and such evidence supports the grandmother cell theory (Barlow, 1995, 2009; Gross, 2002). This paper builds on those previous ones and provides further evidence for widespread use of localist representation by examining columnar organization of the neocortex and the evidence for category cells.

### Other Characteristics of Distributed Representation

(a) **Representational efficiency –** Distributed representation is computationally attractive because it can store multiple concepts using a small set of neurons. With *n* binary output neurons, it can represent *2^n^* concepts because that many different patterns are possible with that collection of binary neurons. With localist representation, *n* neurons can only represent *n* concepts. In Section “Columnar Organization in the Neocortex,” I explain that this property of distributed representation could be its greatest weakness because such a representation cannot be a feasible structure for processing in the brain, given the evidence for columnar organization of the neocortex.
(b) **Mapping efficiency –** Distributed representation allows for a more compact overall structure (mapping function) from input nodes to the output ones and that means less number of connections and weights to train. Such a mapping function requires less training data and will generalize better.
(c) **Resiliency** – A distributed representation based mapping function is resilient in the sense that degradation of a few elements in the network structure may not disrupt or effect the overall performance of the structure.
(d) **Sparse distributed representation –** A distributed representation is sparse if only a small fraction of the *n* neurons is used to represent a subset of the concepts. Some argue that representation in the brain is sparse (Földiak, 1990; Olshausen and Field, 1997; Hromádka et al., 2008; Yu et al., 2013).

McClelland et al. (1995), however, have argued that sparse distributed representation doesn’t generalize very well and that the brain uses it mainly for episodic memories in the hippocampus. They also argue that dense distributed representation is the only structure that can generalize well and that the brain uses this dense form of representation in the neocortex to learn abstract concepts. Bowers (2009) summarizes this particular theory of McClelland et al. (1995) in the following way: “*On the basis of this analysis, it is argued that sparse coding is employed in the hippocampus in order to store new episodic memories following single learning trials, whereas dense distributed representations are learned slowly and reside in cortex in order to support word, object, and face identification (among other functions), all of which require generalization (e.g., to identify an object from a novel orientation)*.” The essence of this theory is that only dense representations can generalize and learn abstract concepts. And thus the only form of distributed representation to consider is the dense one.

### Distributed Processing vs. Distributed Representation

**FIGURE 1:**
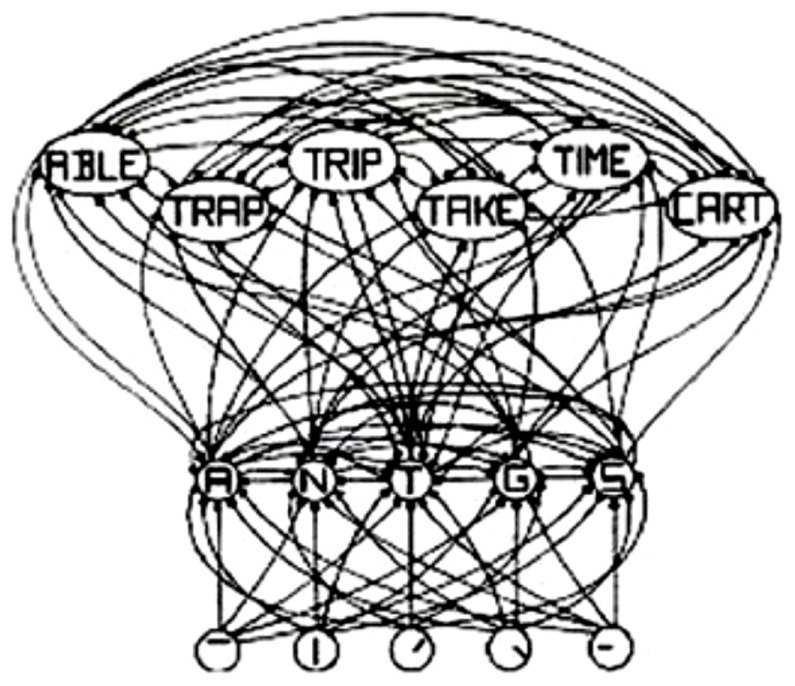
**Schematic diagram of a small subcomponent of the interactive activation model.** Bottom layer codes are for letter features, second layer codes are for letters, and top layer codes are for complete words, all in a localist manner. Arrows depict excitatory connections between units; circles depict inhibitory connections. Adapted from Figure 3 of McClelland and Rumelhart (1981), by permission of American Psychological Association.

The interactive activation (IA) model of McClelland and Rumelhart (1981), shown in **Figure 1**, is a classic localist model. The IA model is a localist model simply because the letter-feature, letter and word units have labels on them, which implies that they have “meaning and interpretation.” Although the model is localist, it uses distributed and parallel processing. For example, all of the letter units are computed in parallel with inputs from the letter-feature layer. Similarly, all of the word units are computed in parallel with inputs from the letter units layer. Thus, both localist and distributed representation can exploit parallel, distributed processing. The representation type, therefore, does not necessarily place a restriction on the type of processing. And localist representation can indeed parallelize computations.

## Columnar Organization in the Neocortex

Although the neocortex of mammals is mainly characterized by its horizontal layers with different cell types in each layer, researchers have found that there is also a strong vertical organization in some regions such as the somatosensory, auditory, and visual cortices. In those regions, the neuronal responses are fairly similar in a direction perpendicular to the cortical surface, while they vary in a direction parallel to the surface (Goodhill and Carreira-Perpiñán, 2002). The set of neurons in the perpendicular direction have connections between them and form a small, interconnected column of neurons. Lorente de Nó (1934) was the first to propose that the cerebral cortex is formed of small cylinders containing vertical chains of neurons and that these were the fundamental units of cortical operation. Mountcastle (1957) was the first to discover this columnar organization (that is, the clustering of neurons into columns with similar functional properties) in the somatosensory cortex of cats. Hubel and Wiesel (1959, 1962, 1968, 1977) also found this columnar organization in the striate cortex (primary visual cortex) of cats and monkeys.

A *minicolumn*, a narrow vertical chain of interconnected neurons across the cortical layers, is considered the basic unit of the neocortex. The number of neurons in these minicolumns generally is between 80 and 100, but can be more in certain regions like the striate cortex. A *cortical column* (or module) consists of a number of minicolumns with horizontal connections. A cortical column is a complex processing unit that receives input and produces outputs. In some cases, the boundaries of these columns are quite obvious (e.g., barrels in the somatosensory cortex and ocular dominance columns in the visual cortex), but not always (e.g., orientation columns in the striate cortex).

**FIGURE 2:**
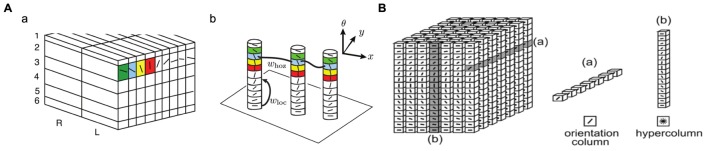
**Orientation columns, ocular dominance columns, hypercolumns, and layers of the striate cortex. **(A)** Adapted from Figure 1 of Bressloff and Carroll (2015).** **(B)** Reprinted from Ursino and La Cara (2004), with permission from Elsevier.

**Figure 2** shows the “ice cube” models that explain the spatial structure of orientation columns, ocular dominance columns and hypercolumns across layers of the striate cortex. An orientation column has cells that have the same orientation (i.e., they respond to an edge or bar of light with the same orientation) and this columnar structure is repeated in the striate cortex for different orientations and different spatial positions [receptive fields (RFs)] on the retina. Tanaka (2003) notes that: “*Cells within an orientation column share the preferred orientation, while they differ in the preferred width and length of stimuli, binocular disparity, and the sign of contrast*.” *Hypercolumn* (*macrocolumn*) cells, on the other hand, respond to the same spatial position (RF) in the retina, but have different orientation preferences. Orientation preferences generally changes linearly from one column to the next, but can have jumps of 90 or 180°. A hypercolumn (macrocolumn) contains about 50–100 minicolumns. According to Krueger et al. (2008), the neocortex has about 100 million minicolumns with up to 110 neurons in each.

**FIGURE 3:**
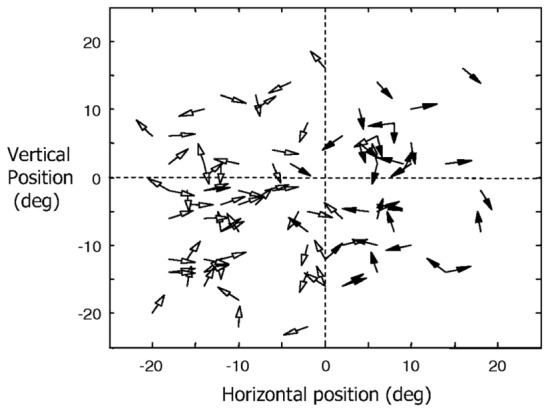
**Distribution of preferred directions for 95 direction-selective LIP neurons of two male rhesus monkeys (filled arrowheads for *monkey H* and open arrowheads for *monkey R*).** Adapted from Figure 6 of Fanini and Assad (2009), by permission of The American Physiological Society.

Direction of motion selectivity columns have been found in the middle temporal (MT) visual area of macaque monkeys (Albright et al., 1984; DeAngelis and Newsome, 1999). **Figure 3** shows the distribution of preferred directions of 95 direction-selective lateral intraparietal area (LIP) neurons of two male rhesus monkeys from the study by Fanini and Assad (2009). Out of the 614 MT direction selective neurons monitored by Albright et al. (1984), 55% responded to moving stimuli independent of color, shape, length, or orientation. The response magnitude and tuning bandwidth of the remaining cells depended on stimulus length, but not the preferred direction. They also found that “*cells with a similar direction of motion preference are also organized in vertical columns and cells with opposite direction preferences are located in adjacent columns within a single axis of motion column*.” Diogo et al. (2002) found direction selective clusters of cells in the visual area MT of the *Cebus apella* monkey that change gradually across the surface of MT but also had some abrupt 180° discontinuities.

**FIGURE 4:**
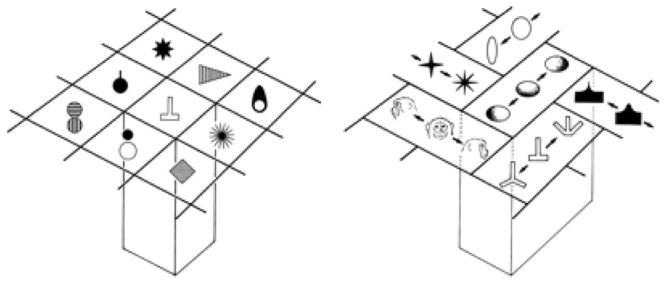
**Columnar modules of region TE.** Adapted from Figures 3 and 7 of Tanaka (2003), by permission of Oxford University Press.

Tanaka (2003) found cells in the inferotemporal cortex (area TE) that selectively respond to complex visual object features and those that respond to similar features cluster in a columnar form. For example, he found cells in a TE column that responded to star-like shapes, or shapes with multiple protrusions in general. Tanaka (2003) notes: “*They are similar in that they respond to star-like shapes, but they may differ in the preferred number of protrusions or the amplitude of the protrusions*.” **Figure 4** shows types of complex objects (complex features) found (or hypothesized) by Tanaka in TE columnar modules. He also notes: “*Since most inferotemporal cells represent features of object images but not the whole object images, the representation of the image of an object requires a combination of multiple cells representing different features contained in the image of the object*.”

In general, neuroscientists have discovered the columnar organization in many regions of the mammalian neocortex. According to Mountcastle (1997), columnar organization is just one form of modular organization in the brain. Mountcastle (1997) notes that the modular structure varies “*in cell type and number, in internal and external connectivity, and in mode of neuronal processing between different large entities.*” DeFelipe (2012) states that “*The columnar organization hypothesis is currently the most widely adopted to explain the cortical processing of information*…” although there are area and species specific variations and some species, such as rodents, may not have cortical columns (Horton and Adams, 2005). However, Wang et al. (2010) found similar columnar functional modules in laminated auditory telencephalon of an avian species (*Gallus gallus*). They conclude that laminar and columnar properties of the neocortex are not unique to mammals. Rockland (2010) states that columns (as modules) are widely used in the brain, even in non-cortical areas.

### Columnar Organization – Its Functional Role and as Evidence for Localist Representation

Neuroscience is still struggling to understand the functional role of columnar organization in cortical processing (Horton and Adams, 2005; DeFelipe, 2012). Here I offer a macro level functional explanation for columnar organization and the way it facilitates fast and efficient processing of information. I also explain why distributed representation (population coding) is inconsistent with and infeasible for the type of superfast processing required in certain parts of the neocortex (and perhaps for other parts of the brain also), where such superfast processing is facilitated by the columnar organization. And columnar organization could be the most extensive neuroscience evidence we have so far for the widespread use of localist representation in the brain.

What the columnar organization reveals is a massively parallel search mechanism – a mechanism that, given an input, searches in parallel for a match within a discrete set of explicitly coded features (concepts). In other words, it tries to match the input, in parallel, to one of the component features in the discrete set, where each such component feature is encoded separately by one or more minicolumns. And the search is parallelized for all similar inputs that arrive simultaneously at a processing stage. That is, each input that arrives at the same time at a processing stage, is processed immediately and separately in a parallel mode. To make this type of parallelized search feasible for multiple inputs, it provides a dedicated macrocolumn (such as a hypercolumn), that encodes the same set of discrete features in its minicolumns, to each and every input (e.g., a RF) so that it can be processed separately in parallel. Horton and Adams (2005) describe a hypercolumn as a structure that contains “*a full set of values for any given set of receptive field parameters*.” The discrete set of explicit features (concepts) – which range from simple features (e.g., line orientation) to complex and invariant ones (e.g., a star-like shape) and where the set of features depends on the processing level – is, of course, learned over time.

Thus, the defining principle of columnar organization is this parallel search for a matching explicit feature within a discrete set, given an input, and performing such searches for multiple inputs at the same time (in parallel), where such parallel searches for multiple inputs are facilitated by deploying separate dedicated macrocolumns for each input. This same parallel search mechanism is used at all levels of processing as necessary. This mode of processing is, without question, very resource intensive. However, this mode of processing is an absolute necessity for the neocortex (and elsewhere in the brain) wherever there is a need for incredibly fast processing.

What’s really unique about columnar organization is the fact that it creates a discrete set of features (concepts) that are explicit. The features are explicit in the sense that they are interpretable and can be assigned meaning. And that organizing principle provides direct evidence for widespread use of localist representation in the cortex and perhaps other areas of the brain (Page, 2000; Roy, 2012, 2013). Here’s an explanation from a computational point of view why columnar organization works that way and why distributed representation, especially dense distributed representation which is hypothesized to be used in the neocortex (McClelland et al., 1995; Poggio and Bizzi, 2004; Bowers, 2009), is not compatible with the processing needs. In dense distributed representation, concepts are coded by means of different patterns of activation across several output units (neurons) of a network. If such a pattern vector, which can code for any number of concepts, is transmitted to another system, that system would have to know how to decode that pattern vector and determine what the concept is. That means that the receiving system would require a decoding processor (a decoder) to understand an incoming pattern vector encoded by signals from a population of neurons. If the columnar organization were to use dense distributed representation to code for features and concepts, it would have to deploy millions of such decoders. That obviously would add layers of processing and slow down the processing of any stimulus. Explicit features, encoded by one or more neurons in cortical columns, make the interpretation (decoding) task simple for subsequent processes. Thus, learning of explicit features by the columnar organization could be mainly about simplification of computations and to avoid a complex decoding problem at every stage of processing.

## Category Cells

There is significant evidence at this point that animal brains, from insects to humans, have the ability to generalize and create abstract categories and concepts and encode and represent them in single cells or multiple cells, where each group of such cells is dedicated to a single category or concept. This reveals a lot about mental representation in the brain. This aspect of abstraction and representation of such abstractions has been ignored and denied in the distributed representation theory.

### The Evidence for Abstract Category Cells

Regarding the ability to create abstract categories, Freedman and Miller (2008) notes (p. 312): “*Categorization is not an ability that is unique to humans. Instead, perceptual categorization and category-based behaviors are evident across a broad range of animal species, from relatively simple creatures like insects to primates*.” Researchers have found such abstraction capability in a variety of studies of animals and insects. Wyttenbach et al. (1996), for example, found that crickets categorize the sound frequency spectrum into two distinct groups – one for mating calls and the other for signals of predatory bats. Schrier and Brady (1987), D’amato and Van Sant (1988) and others have found that monkeys can learn to categorize a large range of natural stimuli. Roberts and Mazmanian (1988) found that pigeons and monkeys can learn to distinguish between animal and non-animal pictures. Wallis et al. (2001) recorded from single neurons in the prefrontal cortex (PFC) of monkeys that learned to distinguish whether two successively presented pictures were same or different. Fabre-Thorpe et al. (1998) found that monkeys can accurately categorize images (food vs. non-food, animal vs. non-animal) with remarkable speed in briefly flashed stimuli. They conclude: “*Overall, these findings imply that rapid categorization of natural images in monkeys must rely, as in humans, on the existence of abstract categorical concepts*.”

Merten and Nieder (2012) found single neurons in the PFC of two rhesus monkeys that encoded abstract “yes” and “no” decisions from judgment about the presence or absence of a stimulus. They note the following (p. 6291): “*we report a predominantly categorical, binary activation pattern of “yes” or “no” decision coding*.” Rolls et al. (1997) found viewpoint-independent spatial view cells in the vicinity of the hippocampus in monkeys. These cells responded when the monkey looked toward a particular view, independent of the place where the monkey is or its head direction. Vogels (1999) found single cells in the anterior temporal cortex of two rhesus monkeys that were involved in distinguishing trees from non-trees in color images. About a quarter of those neurons responded in a category-specific manner (that is, either trees or non-trees). And the responses were mostly invariant to stimulus transformation, e.g., to changes in position and size.

Lin et al. (2007) report finding “nest cells” in the mouse hippocampus that fire selectively when the mouse observes a nest or a bed, regardless of the location or the environment. For example, they found single cells that drastically increased the firing rate whenever the mouse encountered a nest. If the mouse looked away from the nest, that single cell became inactive. In testing for invariance, they note (p. 6069): “*Together, the above experiments suggest that the responses of the nest cell remained invariant over the physical appearances, geometric shapes, design styles, colors, odors, and construction materials, thereby encoding highly abstract information about nests. The invariant responses over the shapes, styles, and materials were also observed in other nest cells*.”

Other single cell studies of the monkey visual temporal cortex have discovered neurons that respond selectively to abstract patterns or common, everyday objects (Fujita et al., 1992; Logothetis and Sheinberg, 1996; Tanaka, 1996; Freedman and Miller, 2008). Freedman and Miller (2008) summarize these findings from single cell recordings quite well (p. 321): “*These studies have revealed that the activity of single neurons, particularly those in the prefrontal and posterior parietal cortices (PPCs), can encode the category membership, or meaning, of visual stimuli that the monkeys had learned to group into arbitrary categories*.”

Different types of faces, or faces in general, represent a type of abstract categorization. Face-selective cells have been a dominant area of investigation in the last few decades. Bruce et al. (1981) were the first ones to find face selective cells in the monkey temporal cortex. Rolls (1984) found face cells in the amygdala and Kendrick and Baldwin (1987) found face cells in the cortex of the sheep. Gothard et al. (2007) studied neural activity in the amygdala of monkeys as they viewed images of monkey faces, human faces and objects on a computer monitor. They found single neurons that respond selectively to images from each category. They also found one neuron that responded to threatening monkey faces in particular. Their general observation is (p. 1674): “*These examples illustrate the remarkable selectivity of some neurons in the amygdala for broad categories of stimuli*.” Tanaka (2003) also observed single cell representation of faces and observes: “*Thus, there is more convergence of information to single cells for representations of faces than for those of non-face objects*.”

On the human side, in experiments with epileptic patients, Fried et al. (1997) found some single medial temporal lobe (MTL) neurons that discriminate between faces and inanimate objects and others that respond to specific emotional expressions or facial expression and gender. Kreiman et al. (2000), in similar experiments with epileptic patients, found MTL neurons that respond selectively to categories of pictures including faces, houses, objects, famous people and animals and they show a strong degree of invariance to changes in the input stimuli. Kreiman et al. (2000) report as follows: “*Recording from 427 single neurons in the human hippocampus, entorhinal cortex and amygdala, we found a remarkable degree of category-specific firing of individual neurons on a trial-by-trial basis*…. *Our data provide direct support for the role of human medial temporal regions in the representation of different categories of visual stimuli.*” Recently, Mormann et al. (2011) analyzed responses from 489 single neurons in the amygdalae of 41 epilepsy patients and found that individual neurons in the right amygdala are particularly selective of pictures of animals and that it is independent of emotional dimensions such as valence and arousal.

In reviewing these findings, Gross (2000) observes: “*Electrophysiology has identified individual neuronsthat respond selectively to highlycomplex and abstract visual stimuli*.” According to Pan and Sakagami (2012), “*experimental evidence shows that the PFC plays a critical role in category formation and generalization*.” They claim that the prefrontal neurons abstract the commonality across various stimuli. They then categorize them on the basis of their common meaning by ignoring their physical properties. These PFC neurons also learn to create boundaries between significant categories.

### Can We Believe these Studies? Are They Truly Category-Selective Cells?

These studies, that claim category-selective response of single cells, are often dismissed because, in these experiments, the cells are not exhaustively evaluated against a wide variety of stimuli. Desimone (1991) responds to that criticism with respect to face cell studies: “*Although they do not provide absolute proof, several studies have tried and failed to identify alternative features that could explain the properties of face cells.*” For example, many studies tested the face cells with a variety of other stimulus, including textures, brushes, gratings, bars and edges of various colors, and models of complex objects, such as snakes, spiders, and food, but there was virtually no response to any such stimulus (Bruce et al., 1981; Perrett et al., 1982; Desimone et al., 1984; Baylis et al., 1985; Rolls and Baylis, 1986; Saito et al., 1986). In fact, each such face cell responded to a variety of faces, including real ones, plastic models, and photographs of different faces (e.g., monkey, human). Rolls and Baylis (1986) found that many face cells actually respond to faces over more than a 12-fold range in the size. Others report that many face cells respond over a wide range of orientations in the horizontal plane (Perrett et al., 1982, 1988; Desimone et al., 1984; Hasselmo et al., 1989). Desimone (1991) concludes: “*Taken together, no hypothesis, other than face selectivity, has yet been advanced that could explain such complex neuronal properties.*”

### Are Category-Selective Cells Part of a Dense Distributed Representation? If So, Do We Need Exhaustive Testing to Find that Out?

A dense distributed representation uses a small set of neurons to code for many different concepts. The basic idea is compressed encoding of concepts using a small physical structure. This also means that different levels of activations of these neurons will code for different concepts. In other words, for any given concept, most of the neurons in such a representation should be active at a certain level. If that is the case and if a so-called “category-selective” cell is actually a part of a dense representation, then stimuli that belong to different abstract concepts should activate the so-called “category-selective” cell quite often. There is no need for exhaustive testing with different stimuli to find that the “category-selective” cell is part of a dense representation. Testing with just a few different types of stimuli should be sufficient to verify that a cell is either part of a dense representation that codes for complex concepts or codes for a lower level feature. And that’s what is usually done in these neurophysiological studies and that should be sufficient. That doesn’t mean that rigorous testing is not required. It only means that we don’t need exhaustive testing to establish that a cell is selective of certain types of stimuli.

## Multisensory Integration in the Brain

Research over the last decade or so has produced a large body of evidence for multisensory integration in the brain and even in areas that were previously thought to be strictly unisensory or unimodal. Ghazanfar and Schroeder (2006) claim that multisensory integration extend into early sensory processing areas of the brain and that neocortex is essentially multisensory. Stein and Stanford (2008) observes that many areas that were previously classified as unisensory contain multisensory neurons. This has been revealed by anatomical studies that show connections between unisensory cortices and by imaging and ERP studies that reveal multisensory activity in these regions. Klemen and Chambers (2012), in a recent article, notes that there is now “*broad consensus that most, if not all, higher, as well as lower level neural processes are in some form multisensory*.” The next two sections examine some specific evidence for multisensory integration.

### The Evidence for Multisensory Integration in Various Parts of the Brain

Neurons in the lateral intraparietal (LIP) area of the PPC are now known to be multisensory, receiving a convergence of eye position, visual and auditory signals (Andersen et al., 1997). Ventral intraparietal area (VIP) neurons have been found to respond to visual, auditory, somatosensory and vestibular stimuli, and for bi- or tri-modal VIP neurons, RFs driven through different modalities usually overlap in space (Duhamel et al., 1998). Graziano et al. (1999) found neurons in the premotor cortex that responded to visual, auditory and somatosensory inputs. Maier et al. (2004) found that the function of these neurons appear to be ‘defense’ related in the sense that monkeys (and humans) are sensitive to visual, auditory and multisensory looming signals that indicate approaching danger. Morrell (1972) reported that up to 41% of visual neurons could be driven by auditory stimuli. Single unit recordings in the IT cortex of monkeys performing a crossmodal delayed-match-to-sample task shows that the ventral temporal lobe may represent objects and events in a modality invariant way (Gibson and Maunsell, 1997). Saleem et al. (2013) recorded from mice that traversed a virtual environment and found that nearly half of the primary visual cortex (V1) neurons were part of a multimodal processing system that integrated visual motion and locomotion during navigation. In an anatomical study, Budinger and Scheich (2009) show that the primary auditory field AI in a small rodent, the Mongolian gerbil, has multiple connections with auditory, non-auditory sensory (visual, somatosensory, olfactory), multisensory, motor, “higher order” associative and neuromodulatory brain structures. They observe that these connections possibly mediate multimodal integration processes at the level of AI. Some studies have shown that auditory (Romanski and Goldman-Rakic, 2002), visual (Wilson et al., 1993; O’Scalaidhe et al., 1999; Hoshi et al., 2000), and somatosensory (Romo et al., 1999) responsive neurons are located within the ventrolateral prefrontal cortex (VLPFC), suggesting that VLPFC is multisensory.

### The Evidence for Modality-Invariant Single Cell Representation in the Brain

Here, I review some of the evidence for modality-invariant single cells in the brain of humans and non-human.

Fuster et al. (2000) were the first to find that some PFC cells in monkeys integrate visual and auditory stimuli across time by having them associate a tone of a certain pitch for 10 s with a color. PFC cells responded selectively to tone and most of them also responded to colors as per the task rules. They conclude that PFC neurons are part of an integrative network that represent cross modal associations. Romanski (2007) recorded from the VLPFC of rhesus macaques as they were presented with audiovisual stimuli and found that some cells in VLPFC are multisensory and respond to both facial gestures and corresponding vocalizations. Moll and Nieder (2015) trained carrion crows to perform a bimodal delayed paired associate task in which the crows had to match auditory stimuli to delayed visual items. Single-unit recordings from the area nidopallium caudolaterale (NCL) found memory signals that selectively correlated with the learned audio-visual associations across time and modality. Barraclough et al. (2005) recorded from 545 single cells in the temporal lobe (upper and lower banks of the superior temporal sulcus (STS) and IT) from two monkeys to measure the integrative properties of single neurons using dynamic stimuli, including vocalizations, ripping paper, and human walking. They found that 23% of STS neurons that are visually responsive to actions are modulated significantly by the corresponding auditory stimulus. Schroeder and Foxe (2002), using intracranial recordings, have confirmed multisensory convergence in the auditory cortex in macaque monkeys. Using single microelectrode recordings in anesthetized monkeys, Fu et al. (2003) confirmed that such convergence in the auditory cortex occurs at the single neuron level.

In some experiments, reported in Quian Quiroga et al. (2009) and others, they found that single MTL neurons can encode an object-related concept irrespective of how it is presented – visual, textual, or sound. They checked the modality invariance properties of a neuron by showing the subjects three different pictures of the particular individual or object that a unit responds to and their spoken and written names. In these experiments, they found a neuron in the left anterior hippocampus that fired selectively to three pictures of the television star Oprah Winfrey and to her written and spoken name (Quian Quiroga et al., 2009, p. 1308). The neuron also fired to a lesser degree to a picture of actress Whoopi Goldberg. And none of the other responses of the neuron were significant, including to other text and sound presentations. They also found a neuron in the entorhinal cortex of a subject that responded (Quian Quiroga et al., 2009, p. 1308) “*selectively to pictures of Saddam Hussein as well as to the text ‘Saddam Hussein’ and his name pronounced by the computer….. There were no responses to other pictures, texts, or sounds*.”

Quian Quiroga (2012, p. 588) found a hippocampal neuron which responded selectively to pictures of Halle Berry, even when she was masked as Catwoman (a character she played in a movie). And it also responded to the letter string “HALLE BERRY,” but not to other names. They also found that a large proportion of MTL neurons respond to both pictures and written names of particular individuals (or objects) and could also be triggered by the name of a person pronounced by synthesized voice. Hence, they conclude: “*These and many other examples suggest that MTL neurons encode an abstract representation of the concept triggered by the stimulus.*” Quian Quiroga et al. (2008) estimate that 40% of MTL cells are tuned to such explicit representation.

Suthana and Fried (2012, p. 428) found an MTL neuron that responded to a picture of the Sydney Opera House but not to 50 other landmarks. It also responded to “*many permutations and physically different representations of the Sydney Opera House, seen in color, in black and white, or from different angles*.” The same neuron also responded to the written words “Sydney Opera.” Nieder (2013) found single neurons in a parieto-frontal cortical network of non-human primates that are selectively tuned to number of items. He notes that: “*Such ‘number neurons’ can track items across space, time, and modality to encode numerosity in a most abstract, supramodal way*.”

## The Existence of A Single Cell-Based Purely Abstract and Layered Cognitive System and Ties to Grounded Cognition

Sections “Category Cells and Multisensory Integration in the Brain” on category cells and multisensory, modality-invariant cells provide significant biological evidence for the existence of a single cell-based purely abstract cognitive system in the brain. The multisensory cells are abstract in the sense that they integrate information from more than one sensory process. And since the multisensory neurons are also present in what are generally considered to be unisensory areas, such an abstract cognitive system is well-spread out in various parts of the brain and not confined to a few areas. This does not mean that cognition in appropriate cases is not grounded in sensory-motor processes (Barsalou, 2008, 2010; Pezzulo et al., 2013). In this section, I extend a well-known abstract model of cognition and show how abstract cognition could be connected to modality-based representations, memory and sensory processes and invoke them as necessary. And it is fair to claim, based on the biological evidence, that both the abstract and non-abstract systems co-exist in the brain and are tightly integrated.

Let’s now examine an often referenced abstract model of cognition from Collins and Quillian (1969) shown in **Figure 5**. Rogers and McClelland (2004, 2008) uses the same model to illustrate how distributed representation might be able to create the same semantic structure. **Figure 5** shows a possible way of storing semantic knowledge where semantics are based on a hierarchy of abstract concepts and their properties. Given the evidence for category and multisensory abstract cells, this model now looks fairly realistic. In this tree structure, nodes represent abstract categories or concepts and arrows reflect properties of that category or concept. For example, the node *bird* has arrows for the properties *feathers. fly*, and *wings*. The arrows point to other nodes that represent these properties, which are also abstract concepts. The semantic tree shows the hierarchical relationship of these abstract concepts and categories. For example, *plant* and *animal* are subcategories of *living thing*. Here, nodes pass down their properties to the descendant nodes. For example, *salmon* inherits all the properties of *fish* (*scales. swim*, and *gills*) and also the properties of *animal* (*move. skin*) and *living thing* (*grow. living*). The properties of higher level concepts reflect the common properties of lower level concepts. The tree produces propositions such as: *living things grow. a plant is a living thing. a tree is a plant*; and *an oak is a tree*. It therefore follows that *an oak can grow*.

**FIGURE 5:**
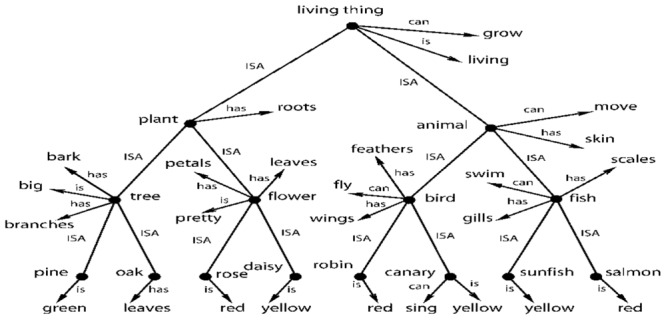
**A taxonomic hierarchy of the type used by Collins and Quillian (1969).** Adapted from Figure 2, Rogers and McClelland (2008) reproduced with permission.

This model can be easily extended to include modality-based representations, memory and sensory processes including simulations. For example, the *robin* node could be a multimodal invariant abstraction that is activated by the physical appearance of a robin (or its picture), by its singing and by the written or spoken name “robin.” However, multisensory integration exists at many levels of processing. For example, there could be a multisensory neuron that integrates information from just the visual and auditory systems. That is, it fires with the physical appearance of a robin (or its picture) and/or when it sings. Many other combinations of sensory information are possible – two at a time, three at a time and so on.

Thus, there could be a layered structure of abstractions in the brain, starting with bi-modals, then tri-modals and so on. And Section “Multisensory Integration in the Brain” cites evidence for such different levels of abstractions. One can think of this layered structure of abstractions in terms of an inverted tree (similar to **Figure 5**) culminating in a single, high-level multimodal abstraction such as the *robin* node of **Figure 5**. Inversely, one can think of the *robin* node having deep extensions into lower levels of modality invariant neurons through an extended tree structure. The lowest level bi-modal invariant nodes, in turn, could be coupled with modal-based representations, memories and sensory processes. A modal representation of a robin in the visual system could have links to a memory system that has one or more generic pictures of robins in different colors and thereby provide access to the imagery part of cognition (Kosslyn et al., 2006). A visual system can also trigger a simulation of the bird flying (Goldman, 2006).

In summary, a purely abstract cognitive system could be tightly integrated with the sensory system and the integration could be through the layered level of abstractions that various multisensory neurons provide. In other words, the conjecture is that a purely abstract cognitive system co-exists with a sensory-based cognition system and perhaps is mutually dependent. For example, the fastest way to trigger the visualization of robins on hearing some robins singing in the background could be through the multisensory (bi-modal) neurons embedded in the sensory systems. The abstract cognitive system could, in fact, provide the connectivity between the sensory systems and be the backbone of cognition in its various forms. So the second part of this Barsalou (2008, p. 618) statement is very consistent with the claims in this section: “*From the perspective of grounded cognition, it is unlikely that the brain contains amodal symbols; if it does, they work together with modal representations to createcognition*.” And Sections “Multisensory Integration in the Brain and The Existence of a Single Cell-Based Purely Abstract and Layered Cognitive System and Ties to Grounded Cognition” answers another Barsalou question (p. 631): “*Can empirical evidence be found for the amodal symbols still believed by many to lie at the heart of cognition*?”

## On the “Meaning and Interpretation” of Single Neuron Response

I come back to the issue of “meaning and interpretation” of the response of a single neuron, an issue that is crucial to the claims of both localist representation and a purely abstract cognitive system. Instead of getting into a philosophical discussion on meaning of the term “meaning,” it would be better if we grounded the discussion in neurophysiology. In neurophysiology, the purpose of testing single neurons with different stimuli is to find the correlation between the response and the collection of stimuli that causes it. This is the “meaning and interpretation” of the response to an external observer such as a scientist. From an internal point of view of the brain, the firing of a neuron can have a cascading effect and trigger other neurons to fire and this generates extra information or knowledge. This is best explained with reference to **Figure 5** and the discussions in Sections “Multisensory Integration in the Brain and The Existence of a Single Cell-Based Purely Abstract and Layered Cognitive System and Ties to Grounded Cognition.” For example, when we see a robin, it would fire a bi-modal neuron that associates the physical appearance of a robin with its singing. This and other multisensory neurons would, in turn, cause the multimodal invariant *robin* node of **Figure 5** to fire. That firing, in turn, would cause the other associated nodes of **Figure 5** to fire, such as the nodes *bird. animal. living thing* and their associated properties. What this means is that the brain activates and collects a body of knowledge after seeing the robin. And that body of knowledge, from multiple cell activations, is the composition of internal meaning of robin in the brain. And that whole body of knowledge can be activated by any and all of the sensory modalities. And that body of knowledge is the sense of “meaning” internal to the brain. And we observe this body of knowledge when we find the multisensory and abstract neurons in the brain. Of course, a simple line orientation cell or a color detection cell may not activate such a large body of abstract knowledge internally in the brain. But these cells still have both internal and external meaning in a similar sense.

## Localist Representation and Symbols

An obvious question is, in what way is localist representation symbolic? I explain it here in a computational sense without getting into a philosophical discussion of symbols. One can think of the neurons, in parts of the brain that use localist representation, as being a unit of memory in a computing system that is assigned to a certain variable. The variables in this case range from a purely abstract concept (e.g., a bird) to something as concrete as a short line segment with a certain orientation. And when any of these neurons fire, it transmits a signal to another processor. These processors could, in turn, be neurons in the next layer of a sensory cortex, in the working memory of the PFC or any other neurons it is connected to. Thus, a localist neuron not only represents a variable in the computing sense, but also does processing at the same time. And, in this computational framework, the so-called variables represented by the localist neurons have meaning inside the brain and are also correlated with stimuli from the external world, as explained in Section “Localist Representation and Symbols.” Hence, these localist neurons are symbols both in the computing sense and because they are correlated with certain kinds of external stimuli.

## No Evidence for Distributed Representation

As mentioned in Section “Other Characteristics of Distributed Representation,” McClelland et al. (1995) have argued that sparse distributed representation does not generalize very well and that the brain uses it mainly for episodic memories in the hippocampus. They also argue that dense distributed representation is the only structure that can generalize well and that the brain uses this dense form of representation in the cortex to learn abstract concepts. And thus the only form of distributed representation to consider is the dense one. But no one has found a dense form of coding anywhere in the brain. In a recent review article, Panzeri et al. (2015) summarize the findings of population coding studies as follows (p. 163): “… *a small but highly informative subset of neurons is sufficient to carry essentially all the information present in the entire observed population*.” They further observe that (pp. 163–164): “*This picture is consistent with the observed sparseness of cortical activity* (Barth and Poulet, 2012) *(at any moment only a small fraction of neurons are active) and is compatible with studies showing that perception and actions can be driven by small groups of neurons* (Houweling and Brecht, 2008).” These observations are also supported by other studies (Olshausen and Field, 1997; Hromádka et al., 2008; Ince et al., 2013; Yu et al., 2013). And these findings are quite consistent with findings on multisensory neurons that indicate that a lot of information can be coded in a compact form by a small set of neurons.

## Conclusion

Neurophysiology has provided a significant amount of information about how the brain works. Based on these numerous studies, one can generalize and claim that the brain uses single cells (or a collection of dedicated cells) to encode particular features and abstract concepts at various levels of processing. One can also claim, based on the evidence for multisensory neurons and category cells, that the brain has a purely abstract and layered cognitive system that is also based on single cell encoding. And that abstract cognitive system, in turn, is connected to the sensory processes and memory. The combined abstract and non-abstract cognitive systems provide the backbone for cognition in its various forms. Parts of the abstract system are also embedded in the sensory systems and provide fast connectivity between the non-abstract systems. This kind of architecture has real value in terms of simplification, concreteness, automation, and computational efficiency. It essentially automates the recognition of familiar patterns at every processing layer and module and delivers such information to other layers and modules in a simplified form.

Cells that encode features and abstract concepts have meaning and interpretation at the cognitive level. Thus, these cells provide easy and efficient access to cognitive level information. Thus far, we have had no clue where cognitive level information was in the brain. These neurophysiological studies are slowly revealing that secret. It could be claimed that these feature and abstract concept cells provide the fundamental infrastructure for cognition and thought.

From these neurophysiological studies, it appears that, at an abstract level, the brain is a massively parallel, distributed computing system that is symbolic. It employs symbols from the earliest levels of processing, such as with discrete sets of feature symbols for line orientation, direction of motion and color, to the highest levels of processing, in the form of abstract category cells and other modality-invariant concept cells.

## Author Contributions

The author confirms being the sole contributor of this work and approved it for publication.

## Conflict of Interest Statement

The author declares that the research was conducted in the absence of any commercial or financial relationships that could be construed as a potential conflict of interest.
